# Effects of metformin therapy on HMGB1 levels in rheumatoid arthritis patients

**DOI:** 10.1186/s40001-023-01476-x

**Published:** 2023-11-15

**Authors:** Lihua Zhang, Yuqing Zhou, Shengzhi Jiang, Yubei Fan, Jierou Huang, Bin Xiao, Hui Rao, Lingyun Huang

**Affiliations:** 1 Department of Rheumatology and Immunology, Hunan Provincial People’s Hospital (The First-Affiliated Hospital of Hunan Normal University), No.89 Guhan Road, Furong District, Changsha, 410016 Hunan People’s Republic of China; 2 The First-Affiliated Hospital of Hunan Normal University (Hunan Provincial People’s Hospital), Changsha, People’s Republic of China

**Keywords:** HMGB1, Rheumatoid arthritis, Metformin, Cytokines, T cell subtypes

## Abstract

**Objective:**

The traditional treatment of rheumatoid arthritis (RA) has some side effects. We aimed to explore the effect of metformin treatment on the expression of HMGB1, cytokines, T cell subtypes and the clinical outcomes in RA patients.

**Methods:**

The present prospective cohort study recruited 124 RA patients (metformin group) who were treated with metformin and conventional therapy (methotrexate, hydroxychloroquine sulfate and sulfasalazine) and 98 RA patients (conventional therapy group) who were only treated with conventional therapy. All subjects were admitted from December 2018 to December 2021 and continuous medication for 90 days. The serum high mobility group box 1 (HMGB1), tumor necrosis factor α (TNF-α), interleukin (IL)-6, IL-1β and C-reactive protein (CRP) levels were measured by enzyme-linked immunosorbent assay (ELISA). Flow cytometric were used to analyze the expression of CD4^+^ and CD8^+^. Demographic and clinical statistics including age, body mass index (BMI), sex, course of disease, erythrocyte sedimentation rate (ESR), rheumatoid factor (RF), visual analogue score (VAS)and disease activity score (DAS)-28 were collected.

**Results:**

The serum levels of HMGB1, CRP, IL-6, CD4+ expression and CD4+/CD8+ ratio were significantly increased in patients with DAS-28 score ≥ 2.6. The serum HMGB1 and cytokines levels of metformin group declined more quickly during the study time. Pearson’s analysis supported that a positive correlation existed between the HMGB1 and IL-6, TNF-α, CRP, CD4^+^, CD4^+^/CD8^+^ ratio, and VAS scores. HMGB1 could be a potential diagnostic biomarker for RA patients in active phase. Serum HMGB1 (95% CI 1.133–1.397, *P* < 0.001) was a factor associated with active RA.

**Conclusion:**

The serum HMGB1 levels were significantly increased in RA patients in active phase. The serum levels of HMGB1 and inflammatory factors and VAS scores were decreased gradually with metformin treatment. HMGB1 might act as a novel therapeutic target for RA.

## Introduction

Rheumatoid arthritis (RA) is a chronic, systemic autoimmune disease characterized by aggressive arthritis [1]. The global prevalence of rheumatoid arthritis is approximately 5 per 1000 adults, which affects women two to three times more often than men [2]. There are many risk factors for RA, including environmental influences [3], lifestyle [4], cigarette smoking [5], hereditary [6], etc. [7]. The conventional treatment regimens (disease-modifying anti-rheumatic drugs (DMARDs) for RA are gradually being standardized [8, 9]. However, more and more RA patients are drug intolerant, and immunosuppressants such as methotrexate and leflunomide have serious side effects (hair loss, liver impairment, bone marrow suppression, etc.), which have limited the use of traditional DMARDs [10–12]. In recent years, metformin has been confirmed to be an effective drug for the treatment of RA [13, 14], but its treatment mechanism needs to be further clarified.

Inflammation in RA patients mainly affects small joints and is characterized by pain and swelling. Inflammatory pathways in the patient's body are activated and the secretion of inflammatory factors increases, exacerbating the patient's symptoms and pain, leading to a decrease in quality of life, physical function and work capacity [15]. Metformin not only improves metabolic indicators to alleviate chronic inflammation, but also possesses direct anti-inflammatory effects [16]. Animal studies have shown that metformin improves various diseases, such as polycystic ovary syndrome (PCOS) and ankylosing spondylitis, through its anti-inflammatory mechanisms [17, 18]. Furthermore, there is evidence indicating that metformin can inhibit the inflammatory response induced by collagen in rat arthritis [19]. High mobility group box 1 (HMGB1) is a highly conservative nucleoprotein in all cell types [20]. Recently, HMGB1 has been found to play multiple roles in the regulation of inflammation and responses to cells and tissues [21]. In addition, HMGB1 mediates inflammatory responses in a variety of diseases and physiological processes by mediating the formation of inflammatory corpuscle and thus activates inflammation-mediated cell death pathways [22, 23]. Previous studies suggested that HMGB1 can activate inflammatory pathways in RA [24], and inhibition of HMGB1 can suppress inflammatory responses in RA animal models [25]. Metformin has been confirmed to inhibit inflammatory response by inhibiting HMGB1 in animal models and cells of RA. However, at present, there is no clinical study to support the above results.

In this prospective observational cohort research, we aimed to explore the serum levels of HMGB1 in RA patients and its correlation with cytokines, T cell subtypes and the clinical outcomes of the patients’. This study may reveal the clinical significance of HMGB1 in RA patients, as well as provide novel research targets for RA treatment.

## Methods

### Subjects

The present prospective cohort study recruited 124 RA patients (metformin group) who were treated with metformin (orally at 0.25 g, twice a day) and conventional therapy (methotrexate, orally at 10 mg, once a week, hydroxychloroquine sulfate, orally at 0.2 g, twice a day, sulfasalazine, orally at 0.5 g, three times a day). We also enrolled 98 RA patients (conventional therapy group) who were only treated with conventional therapy. All subjects were admitted from December 2018 to December 2021 and continuous medication for 90 days. Patients were diagnosed with RA according to the 2010 rheumatoid arthritis classification criteria by the American College of Rheumatology/European League Against Rheumatism [26]. The exclusion criteria included: (1) patients with other autoimmune diseases; (2) patients with diabetes; (3) patients with serious infection, severe liver or renal dysfunctions, malignancy, or cardiovascular dysfunctions; (4) patients who dropped out of the study due to drug intolerance, hypoglycemia, or other reasons in the metformin treatment group. The disease activity score (DAS) 28 was used to evaluate patient's disease activity, including patients in the active phase (DAS-28 score < 2.6) and patients in remission (DAS-28 score ≥ 2.6).

All patients were followed up for 90 days. Written informed consent was obtained from all participants. This research has obtained approval from the ethics committee of Hunan Provincial People’s Hospital.

### Blood sampling measurement

The serum HMGB1, tumor necrosis factor α (TNF-α), interleukin (IL)-6, IL-1β and C-reactive protein (CRP) levels were measured by enzyme-linked immunosorbent assay (ELISA). Blood samples of fasting cubital venous (5 mL) were collected within 24 h after admission for all cases. Samples were centrifuged at 2000*g* for 15 min, followed with ELISA tested using commercially available kits (HMGB1 MBS7722054 MyBioSource, IL-6 MBS175877 MyBioSource, CRP MBS8123937 MyBioSource, TNF-α MBS824943 MyBioSource). All these inflammatory factors were measured when the RA patients were just hospitalized and the 7, 14, 28, and 90 days during the treatment.

### Flow cytometric analysis

5 ml of peripheral elbow vein blood was collected from all subjects at admission and 90 days of admission, and peripheral blood mononuclear cells were isolated as described elsewhere [27, 28]. CD4^+^ and CD8^+^ expression was measured using flow cytometry. After centrifugation (1500 rpm × 5 min), 200 μL of the above cell suspension was added to pre-chilled PBS, washed 2–3 times, and then the cells were resuspended in 100 μL of PBS solution. Add 5 μL of PE-Anti-Human CD4 (MBS2569691, MyBioSource) and FITC-Anti-CD8 (MBS9461514, MyBioSource) to the centrifuge tube. Then, incubated for 2 h at 4 °C and centrifuged again (3000 rpm × 10 min). Using FACS Calibur flow cytometer (BD Biosciences, USA) with Diva software (version 6.1, BD Pharmingen USA) to measure the expression of CD4^+^ as well as CD8^+^.

### Data collection and scale scoring

Demographic and clinical statistics including age, body mass index (BMI), sex, course of disease, etc., were collected. Using an automatic biochemical analyzer to perform whole blood test by Hitachi 7600 of Hitachi Corporation, and erythrocyte sedimentation rate (ESR) was recorded. Rheumatoid factor (RF) was detected by immune turbidimetry. The visual analogue score (VAS) was recorded to assess the severity of the patient’s condition and recovery.

### Statistical analysis

Data were expressed by mean ± SD or median (range) according to distribution, which was confirmed by Kolmogorov–Smirnov analysis. Mann–Whitney test or Student’s *t* test was used for comparison between two groups. Kruskal–Wallis test or one-way analysis of variance (ANOVA) followed by Tukey’s post hoc test was used for comparison among three or more groups. Chi-square test was used for rates. Pearson’s rank correlation was used for correlation analysis. Logistic regression was performed for risk factors of patients in active phase. *P* < 0.05 regarded a significant difference. All data used SPSS 18.0 for statistical analyses.

## Results

### Clinical characteristics of all participants

This study enrolled 222 RA patients. All subjects were divided into two groups including metformin group (*n *= 124) and conventional therapy group (*n *= 98). The clinical characteristics of all participants are shown in Table 1.

**Table 1:** Basic characteristics of all patients

Variable	Metformin group, *n *= 124	Conventional therapy group, *n *= 98	*p*
Age, y	51.5 (38–65)	52 (31–69)	0.492
Sex, female (%)	91 (73.39)	71 (72.45)	0.999
BMI	25.39 ± 2.22	25.22 ± 2.24	0.577
course of disease, y	4 (1–9)	5 (1–10)	0.345
ESR (mm/h)	29.01 ± 16.24	26.82 ± 15.78	0.312
RF (IU/ml)	160.03 ± 107.40	160.71 ± 108.26	0.983
VAS score	3.9 (0.7–5.8)	4.1 (0.8–5.7)	0.878
CRP (pg/ml)	127.08 ± 26.43	128.29 ± 27.27	0.738
IL-6 (pg/ml)	32.82 ± 9.93	33.39 ± 10.31	0.675
TNF-α (pg/ml)	53.21 ± 5.36	53.83 ± 5.03	0.382
HMGB1 (pg/ml)	85.49 ± 16.34	84.56 ± 14.56	0.662
DAS-28 score	3.02 (0.74–5.53)	3.08 (0.70–5.45)	0.901

### Expression of HMGB1, inflammatory factors and T cell subtypes in RA patients

**Fig. 1:**
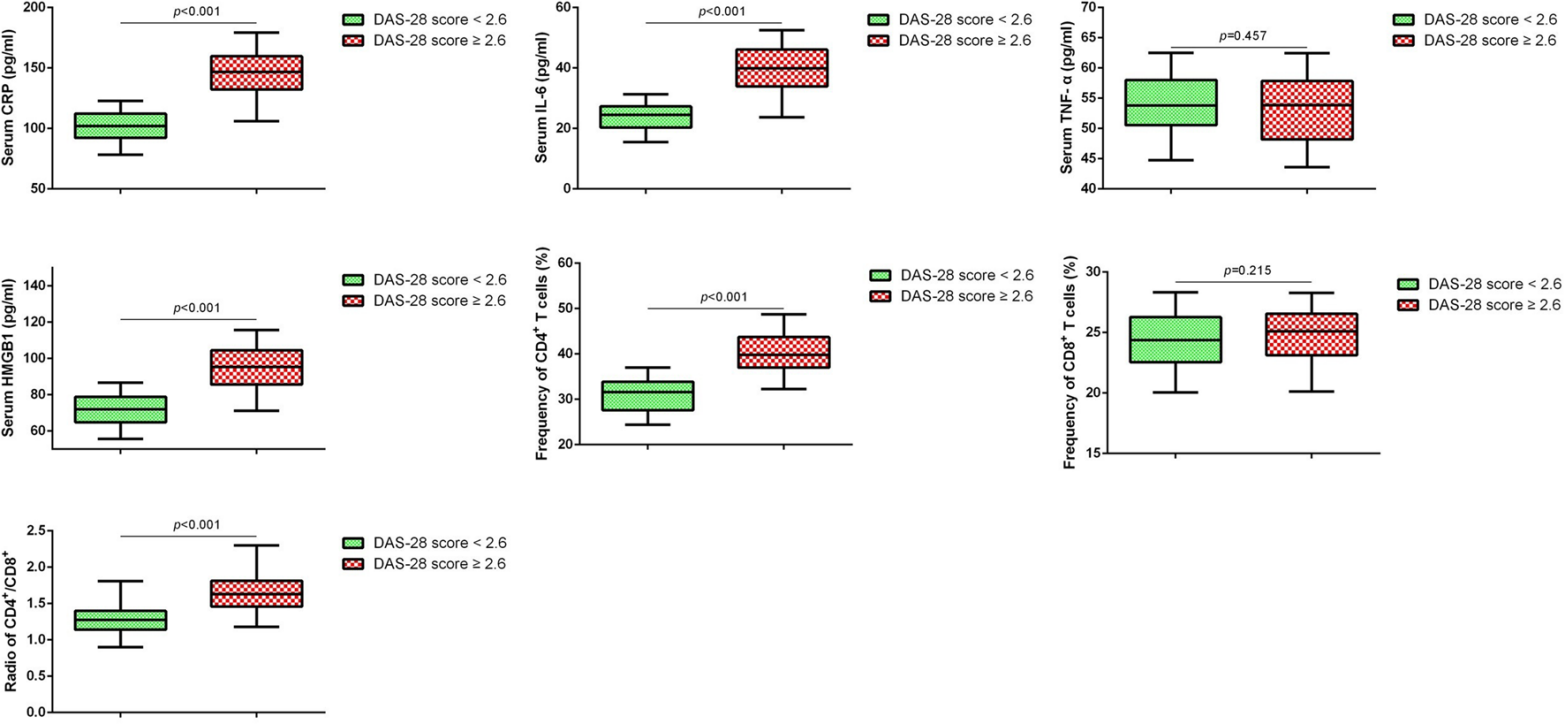
Expression of HMGB1, inflammatory factors and T cell subtypes in RA patients

Then, we measured the expression of HMGB1, inflammatory factors, CD4^+^ and CD8^+^ in RA patients. As shown in Fig. 1, the serum levels of HMGB1, CRP, IL-6, CD4^+^ expression and CD4^+^/ CD8^+^ ratio were significantly increased in patients with DAS-28 score ≥ 2.6 (*p* < 0.05).

### Effect of metformin treatment on serum HMGB1, inflammatory factors levels and T cell subtypes in RA patients

**Fig. 2:**
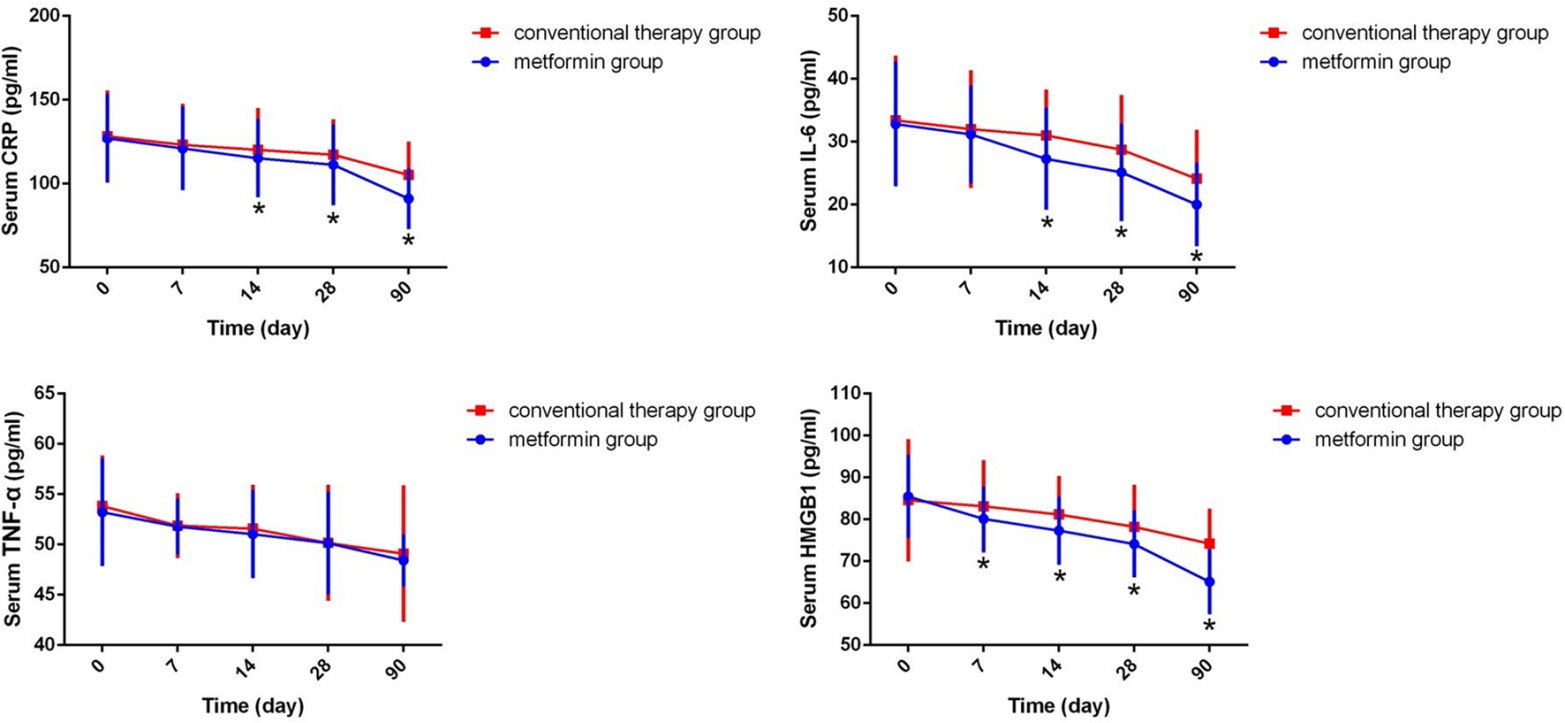
Comparisons of HMGB1 and inflammatory factors between two groups**.** *P < 0.05 compared between metformin group and conventional therapy group

To further investigate the relationship between HMGB1 and inflammation in RA patients, we draw line graphs of all subjects to show the dynamic variations of all cytokines. It was shown that the HMGB1 and inflammatory factors levels were decreased gradually with the treatment in both groups (Fig. 2). We found no obvious differences in the serum HMGB1, TNF-α, CRP and IL-6 levels between two groups when the patients just hospitalized. However, the serum HMGB1 and cytokines levels of metformin group declined more quickly during the study time (*p* < 0.05).

**Fig. 3:**
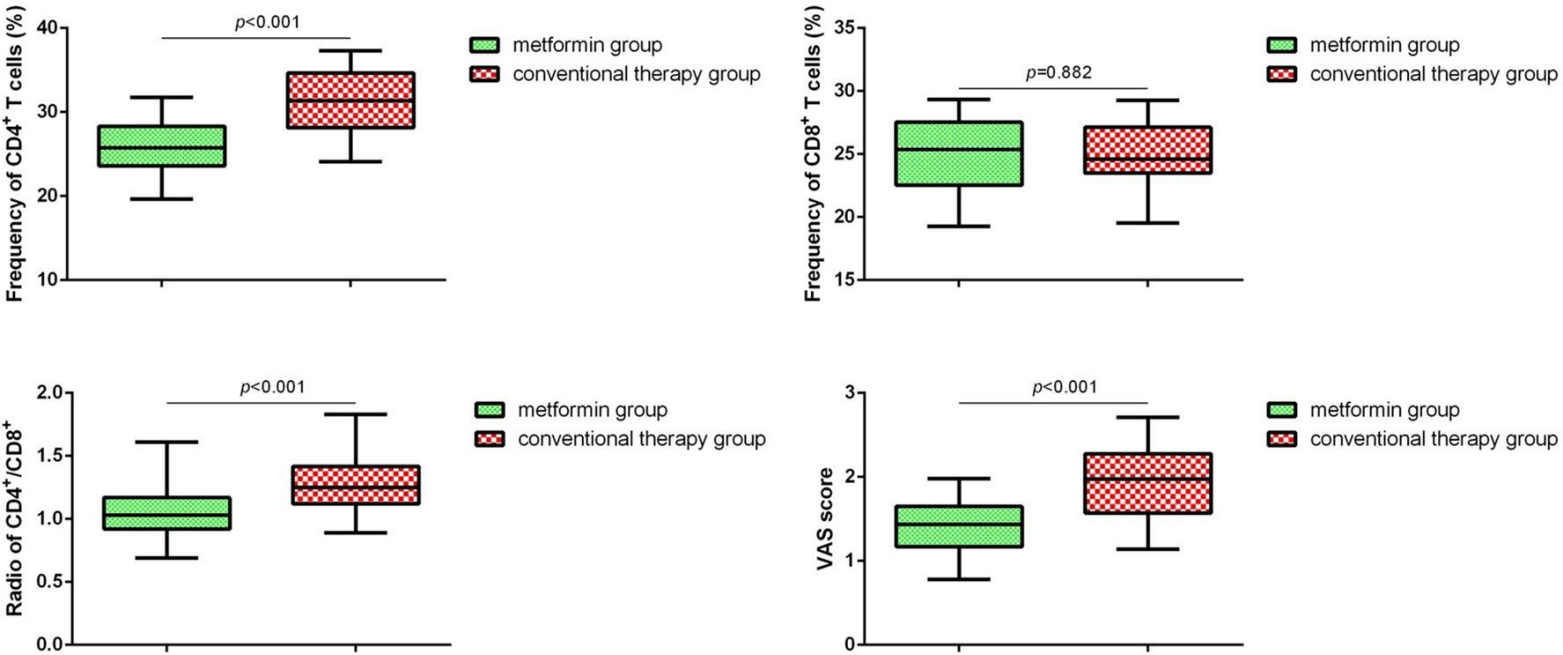
VAS scores and the expression of T cell subtypes in RA patients after metformin treatment

In addition, we detected CD4^+^, CD8^+^ expression and VAS scores of patients after 90 days of the treatment. Compared with conventional therapy group, we found that the expression of CD4^+^, CD4^+^/CD8^+^ ratio and VAS scores were remarkably decreased in metformin group (Fig. 3, *p* < 0.05). Pearson’s analysis supported that a positive correlation existed between the HMGB1 and IL-6, TNF-α, CRP, CD4^+^, CD4^+^/ CD8^+^ ratio, VAS scores (Table 2).

**Table 2:** Correlation analysis among HMGB1, inflammatory factors and clinical outcomes

Variables	HMGB1	
	Pearson’s correlation	*p*
IL-6	0.602	< 0.001
CRP	0.595	< 0.001
TNF-α	− 0.010	0.881
CD4^+^	0.583	< 0.001
CD8^+^	0.098	0.147
CD4^+^/CD8^+^	0.479	< 0.001
VAS scores	0.676	< 0.001

### Diagnostic value of HMGB1 for RA patients in active phase

**Fig. 4:**
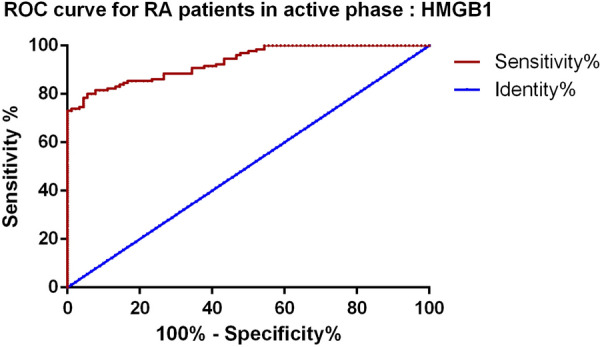
ROC curves for HMGB1 in diagnostic of RA patients in active phase

Furthermore, we draw ROC curves to assess the diagnostic value of HMGB1 for RA patients in active phase. The result showed that HMGB1 could be a potential diagnostic biomarker for RA patients in active phase (Fig. 4), the AUC of HMGB1 was 0.932, cutoff value 80.21 pg/ml, sensitivity 83.8%, specificity 85.9%.

### Risk factors of RA patients in active phase by logistic regression analysis

The risk variables for RA patients in active phase were calculated using binary regression analysis. Both univariate and multivariate logistic regression analyses were conducted to calculate the risk variables in patients with active RA. The results of the univariate analysis showed that ESR, RF, CRP, IL-6, CD4 +, CD4+/CD8+, and HMGB1 were factors associated with active RA (Table 3). Multivariate analysis established two models: Model 1 (age, BMI, disease duration, ESR, RF) and Model 2 (CRP, IL-6, TNF-α, CD4+, CD8+, CD4+/CD8+, HMGB1) and the analysis results indicated that ESR, RF, IL-6, CD4+, CD4+/CD8+, and HMGB1 were factors associated with active RA.

**Table 3:** Risk factors of RA patients in active phase by logistic regression analysis

Variables	Univariate			Multivariate		
	OR	95% CI	P	OR	95% CI	P
				Model 1		
Age	1.018	0.986–1.052	0.274	0.979	0.902–1.063	0.611
BMI	1.051	0.931–1.185	0.421	0.970	0.702–1.341	0.855
Course of disease	1.020	0.912–1.141	0.727	1.136	0.862–1.496	0.365
ESR	1.145	1.103–1.188	< 0.001	1.184	1.095–1.281	< 0.001
RF	1.051	1.034–1.069	< 0.001	1.066	1.033–1.099	< 0.001
				Model 2		
CRP	1.258	1.161–1.363	< 0.001	1.551	1.110–2.169	0.100
IL-6	1.599	1.382–1.851	< 0.001	1.985	1.244–3.169	0.040
TNF-α	0.981	0.931–1.032	0.455	0.892	0.688–1.156	0.388
CD4^+^	2.061	1.674–2.537	< 0.001	1.865	1.447–2.380	< 0.001
CD8^+^	1.082	0.956–1.224	0.214	1.053	0.760–1.460	0.755
CD4^+^/CD8^+^	1123.75	288.356–8775.293	< 0.001	1793.381	252.208–12,780.682	< 0.001
HMGB1	1.236	1.167–1.309	< 0.001	1.258	1.133–1.397	< 0.001

## Discussion

Irregular treatment leads to joint deformity and loss of function can lead to a decrease in the patient's body, quality of life and social participation, bringing a huge economic burden to the patient's family and society [29]. Therefore, it is urgent to develop new biomarkers and comprehensive approaches to evaluate the patient's condition, as well as explore new therapeutic targets. In this research, our finding indicated that metformin treatment effectively reduced serum HMGB1 levels, and serum HMGB1 was a factor associated with active RA.

Metformin acts on a variety of intracellular signaling pathways, exerting immunomodulatory and anti-inflammatory effects by controlling the inflammatory response and the activation and differentiation of T and B cells [30]. Metformin exerted anti-inflammatory effects by inhibiting catabolic products and blocking HMGB1 translocation [31]. Sun et al*.* suggested metformin improved LPS-inhibited cellular autophagy by targeting HMGB1 via the AMPK/mTOR pathway [32]. These results indicated that metformin could inhibit inflammatory responses in animals and cells by mediating HMGB1. In our clinical study, we also found that serum HMGB1 levels and VAS scores were remarkably decreased with metformin treatment. This suggested that HMGB1 might play a significant role in the progression of RA, prompting us to further investigate biomarkers for active RA. Several biomarkers have been used to assess or diagnose RA. Zhang et al*.* suggested that regulatory T cells (Tregs) and interleukin-35 (IL-35) were decreased in RA patients and negatively correlated with ESR and DAS28 [33]. A cross-sectional study by Nakhjavani et al*.* confirmed the serum YKL-40 level of RA patients was significantly elevated than that of healthy controls and YKL-40 could be used to evaluate the activity of RA [34]. Soleimani et al*.* supported the serum glucose-6-phosphate isomerase (G6PI) could be used as a diagnostic marker for RA [35]. In our study, we found that the serum HMGB1 levels were elevated in activity-phase RA patients and could be a potential diagnostic biomarker for RA patients in active phase.

HMGB1, as a new inflammation related factor, has been confirmed to produce a marked effect in multiple diseases. Several in vivo studies have found that HMGB1 mediates inflammatory responses by regulating the ratio of CD4^+^ and CD8^+^ T cells to activate inflammatory pathways [36, 37]. Our findings were similar to these studies, we also found a positive correlation existed between the HMGB1 and CD4+, CD4+/CD8+ ratio in this study. In addition, previous clinical studies have shown the clinical significance and prognostic role of HMGB1 in pneumonia, cholecystitis and pancreatitis [38–40]. Modulation of the HMGB1 signaling pathway can suppress cytokine expression, alleviate cardiomyocyte apoptosis, and reduce fibrosis in RA model [25]. Targeting HMGB1 can improve the prevalent destructive events in RA [41]. In the present study, we also observed that HMGB1 levels were associated with disease activity and clinical outcomes, which was consistent with the results of Pullerits et al. [42]. However, Ozturk et al*.* found that the serum HMGB1 levels were no difference in septic and non-septic arthritis, which might be related to different study populations and disease types [43]. More prospective clinical studies are still needed in the future to validate our conclusions.

This present research also has some limitations. First, we only included a small size of study population and it is a single-center study. Secondly, we only checked a small number of inflammatory factors and T cells subtypes. Finally, the molecular mechanism of HMGB1 affecting RA development is unclear.

## Conclusion

This study showed that the serum HMGB1 levels were significantly increased in RA patients in active phase. The serum levels of HMGB1 and inflammatory factors and VAS scores were decreased gradually with metformin treatment. Pearson’s analysis supported that a positive correlation existed between the HMGB1 and IL-6, TNF-α, CRP, CD4+, CD4+/CD8+ ratio, VAS scores. HMGB1 could be a potential diagnostic biomarker for RA patients in active phase and might act as a therapeutic target for RA.

## Data Availability

All data can be requested from the authors.
