# Correction: Effects of metformin therapy on HMGB1 levels in rheumatoid arthritis patients

**DOI:** 10.1186/s40001-023-01573-x

**Published:** 2023-12-16

**Authors:** Lihua Zhang, Yuqing Zhou, Shengzhi Jiang, Yubei Fan, Jierou Huang, Bin Xiao, Hui Rao, Lingyun Huang

**Affiliations:** 1 Department of Rheumatology and Immunology, Hunan Provincial People’s Hospital (The First-Affiliated Hospital of Hunan Normal University), No.89 Guhan Road, Furong District, Changsha, 410016 Hunan People’s Republic of China; 2 The First-Affiliated Hospital of Hunan Normal University (Hunan Provincial People’s Hospital), Changsha, People’s Republic of China

**Correction: European Journal of Medical Research (2023) 28:512** 10.1186/s40001-023-01476-x

In the original publication of the article, the “Ethics approval and consent to participate” statement was incorrectly given as “All patients signed the informed consent. The present study obeyed Declaration of Helsinki. Study approval was obtained by the ethical committee of the authors’ afliation (No. HPHP-2018-0095)”. The corrected statement should read as “All patients signed the informed consent. The present study obeyed the Declaration of Helsinki. Study approval was obtained by the ethical committee of Hunan Provincial People’s Hospital [2023]-59”. The original article [1] has been corrected.
